# Prevalence and associated factors of alcohol use patterns among university students in Uganda

**DOI:** 10.11604/pamj.2020.37.339.21136

**Published:** 2020-12-14

**Authors:** Louis Henry Kamulegeya, Peter James Kitonsa, Eric Okolimong, Gloria Kaudha, Sonia Maria, Etheldreda Nakimuli-Mpungu

**Affiliations:** 1 The Medical Concierge Group, Kampala, Uganda,; 2 College of Health Sciences, Makerere University, Kampala, Uganda,; 3 Infectious Diseases Institute, Kampala, Uganda,; 4 Uganda Heart Institute, Mulago Hospital Complex, Kampala, Uganda

**Keywords:** Alcohol misuse, heavy episodic drinking, stress, depression, university students

## Abstract

**Introduction:**

majority of alcohol use pattern studies among university students are from developed countries. Information about the different alcohol use patterns and their correlates among university students in sub-Saharan Africa is limited. The aim of this study was to examine the prevalence and cardinal demographic and psychosocial factors associated with specific alcohol use patterns among Ugandan university students.

**Methods:**

a cross section study conducted over 5-months among university students using a standardized socio-demographic questionnaire screened for alcohol use problems, depression symptoms and academic stress using the alcohol use disorders identification test (AUDIT), self-reporting questionnaire (SRQ-20) and the higher education stress inventory (HESI) respectively. Multivariate multinomial regression models were used to determine factors independently associated with a specific alcohol use pattern with low-risk drinkers as the reference group.

**Results:**

a thousand out of 1200 students completed all study requirements for which 60% were males; median age was 22.3 (SD=2.36). The prevalence estimates of any alcohol use, low-risk drinking, heavy episodic drinking and alcohol misuse were 31%, 17.3%, 4.5% and 8.9% respectively. In comparison to low-risk drinkers, students reporting heavy episodic drinking were more likely to report high levels of academic stress (P-value <0.10). Those with alcohol misuse were more likely to be males and with significant depression symptoms (P-value ≤0.05). Non-alcohol users were more likely to report high levels of academic stress (P-value ≤0.05).

**Conclusion:**

the prevalence of maladaptive alcohol use patterns is high among Ugandan university students. Integrating peer led psychological interventions into student health services is desperately needed.

## Introduction

The harmful use of alcohol is a serious global health burden causing both acute and chronic health problems and adverse social consequences are common when they are associated with alcohol consumption. Every year, the harmful use of alcohol kills 2.5 million people, including 320,000 young people between 15 and 29 years of age [1]. It is the third leading risk factor for poor health globally and harmful use of alcohol was responsible for almost 4% of all deaths in the world, according to the estimates for 2018 [1].

University students have been reported to consume higher levels of alcohol than non-university students worldwide [2]. Various theories have been advanced to explain this observation. For example, the tension reduction theory contends that tension producing circumstances (i.e. stressors) could lead to increased drinking [3,4]. Given that alcohol is perceived to reduce tension, high levels of stress and depressive symptoms are associated with alcohol consumption [5-8]. Indeed, college students have been reported to consume alcohol to potentially relax or relieve tension, celebrate, feel comfortable with the opposite sex, as a reward for working hard and to get away from troubles [9].

Unfortunately, excess consumption of alcohol has adverse physical and mental health consequences which lead to impaired social work, interpersonal and academic malfunctioning. The majority of research studies on the alcohol use patterns of university students have been conducted in developed countries. These studies have shown that among university students, factors including year of study, peer influence, age, having an income source among others as divers to high levels of alcohol consumption in these settings [10].

University life is a developmental transition to new responsibilities in absence of well-established networks of social support. On the other hand, it also represents freedom, liberty and fewer restrictions due to living away from parents [11]. Both aspects can increase the use of alcohol among university students. The alcohol patterns of young adults vary according to gender in the same way as in the general population [5]. In general, men drink more alcohol and experience more and different kinds of alcohol related problems. To our knowledge, alcohol use patterns among university students in sub-Saharan Africa are limited and non-existent in Uganda. Studies on alcohol consumption among university students have mainly focused on prevalence rates and associated factors of alcohol use problems [12,13]. Information about the different alcohol use patterns and their correlates is lacking. Further, the extent to which various factors such as gender and year of study may be associated with various alcohol use patterns has not been documented in Uganda.

In the present report, we examine the prevalence and cardinal demographic and psychosocial factors associated with low-risk drinking, heavy episodic drinking (binge drinking) and alcohol misuse (probable abuse and dependence) among university students in Uganda. Information from the study can be used to guide the development of student-led group interventions to prevent alcohol use problems and their adverse consequences among university students in Uganda.

## Methods

**Ethics statement:** participation in the study was voluntary and anonymous. Written informed consent was obtained from all study participants. They were also informed that they could terminate the participation at any point while filling out the questionnaire. The permission to conduct the study was granted by the Makerere University School of Bio-Medical Sciences Institutional Review Board and the Uganda National Council of Science and Technology.

**Sample:** the analysis described in this paper was based on data collected among university students in Uganda as part of the cross-sectional study investigating the psychological well-being, academic stress, alcohol use problems among Makerere University students. The participating university was selected on basis of personal contacts of the researchers. The university is situated in an urban environment and alcohol consumption was not restricted on the campuses. A self-administered questionnaire was distributed to participants drawn from all the 10 colleges of the university: business and management, agriculture and environmental science, engineering, design art and technology, education and external studies, health sciences, humanities and social sciences, computing and information science, natural sciences, veterinary medicine and biosciences, and school of law, during regular classes of courses which were selected in order to obtain about one third of the sample. The overall response rate was 83% and the final sample included 1,000 students. In order to increase the accuracy of self-reports, students were assured that their answers would remain confidential and data protection was observed at all times.

**Study procedure:** over a five-month period (August-December 2012), university students were randomly approached and asked to participate in a study investigating the psychological well-being, academic stress and alcohol use problems among university students. The eligibility criteria required participants to be aged 18 years or older, who had the ability to comprehend study procedures and provide informed consent. Individuals with hearing or visual impairment were ineligible for the study. The students were approached by the trained research assistants who explained study procedures and then obtained informed consent. Students who provided informed consent were then asked to complete a standardized questionnaire, for which they received no compensation. The questionnaire was developed in English, the official language of Uganda, since the target populations were university students who belong to different ethnic backgrounds.

### Study measures

**Covariates:** a number of covariates were assessed using the standardized structured questionnaire.

**Socio-demographic variables:** the questionnaire asked about descriptive information including age, gender, marital status, employment status and years spent at the university. Gender was coded as 1 (male) and 0 (female). Age was categorized and coded as 0 (less than 21 years), 1 (21-25 years) and 2 (above 25 years). Employment status was categorized and coded as 1 (employed) and 0 (unemployed). Marital status was categorized and coded as 1 (single) and 0 (married). Years spent at the university were coded as 1 (one year), 2 (two years), 3 (three years), 4 (four years) and 5 (five years).

**Type of scholarship:** students were asked if they had received a government scholarship (coded 1) or had obtained a private scholarship (coded as 0).

**Psychosocial variables:** depressive symptoms were assessed using the self-reporting questionnaire (SRQ-20). This brief, time- and cost-efficient questionnaire is recommended by the World Health Organization for screening common mental disorders such as depression and anxiety in developing countries. It has been successfully translated into at least 20 languages in several developing countries, with acceptable measures of reliability and validity [14,15]. The time span for each item refers to the individual´s feelings over the past 30 days. A score of 1 indicates that the symptom was present during the past month; a score of 0 indicates the symptom was absent, with a maximum possible score of 20.

Academic stress was assessed using the higher education stress inventory (HESI), a comprehensive instrument, consisting of 33 items positively and negatively worded with regard to stressors [16]. Each item is rated on a four-point Likert scale, 1-4, (totally disagree, somewhat disagree, somewhat agree, totally agree). Positively worded items have reversed scoring, so high scores always denote high stress. A variable indicating academic stress level was created and categorized as total scores ≥66 (coded 1) and total scores <66 (coded as 0).

**Suicidal ideation:** students were asked if they had felt fed up with their lives in the past 12 months. Positive responses were coded 1 and negative responses were coded 0.

**Mental health service use:** students were asked if they had consulted a psychologist, counselor or psychiatrist in the past 12 months. Positive responses were coded 1 and negative responses were coded 0.

### Outcome variables

**Alcohol use patterns:** in order to gather data on alcohol use patterns, we included the alcohol use disorders identification test (AUDIT) [17,18]. The AUDIT was developed by the World Health Organization (WHO) as a simple method of screening for excessive alcohol consumption in the past 12 months [17]. It consists of 10 questions on recent alcohol use (items 1-3), alcohol dependency syndromes (items 4-6) and alcohol-related problems (items 7-10). Each of the 10 questions is rated on a four-point scale. The total score ranges from 0 to 40. For the purpose of this analysis, students were divided into 4 groups based on AUDIT scores and their pattern of alcohol consumption during the prior 30 days: non-drinkers, low-risk drinking, heavy episodic drinking and alcohol misuse. Non-drinkers were those who did not drink alcohol in the previous 30 days. Low-risk drinkers were those who consumed at least 1, but fewer than 5 drinks on any one occasion and had AUDIT scores less than 8. Heavy episodic drinkers were defined as those who consumed 5 or more drinks per occasion and had an AUDIT scores less than 8. Problem drinkers were defined as those who had an AUDIT scores greater than 8.

**Statistical analyses:** the goal of the analyses was to estimate and identify, among university students, the prevalence and factors associated with any alcohol use, abstaining from alcohol use, low-risk drinking, heavy episodic drinking and problem drinking. Initially, a binary variable was created for alcohol use depression, with the variable coded 1 for any alcohol use and coded 0 for no alcohol use. We used simple logistic regression models to evaluate socio-demographic and psychosocial variables that were significantly correlated with any alcohol use. Variables significant at P-value ≤0.20 in the unadjusted analysis were included in the final multiple logistic regression analysis. Both forward and backward selection of variables was carried out using this final model.

Thereafter, alcohol use patterns were treated as a categorical variable with four categories including those who never used alcohol, those who were low-risk drinkers, those who were heavy episodic drinkers and those who were problem drinkers. Multinomial regression was used to calculate the unadjusted relative odds of various variables predicting being in group 1 (never used alcohol) relative to being in group 2 (low-risk drinkers-the reference group), predicting being in group 3 (heavy episodic drinkers) relative to being in group 2 (the reference group) and predicting being in group 4 (problem drinkers) relative to being in group 2 (the reference group).

Independent variables significant at p-value ≤0.2 in the unadjusted analysis were included in the final multinomial regression analysis. The same independent variables were used in each regression model. In the final model, the odds ratios represented the adjusted relative odds of various variables predicting the absence of alcohol use (group 1) relative to low-risk drinking (group 2), the presence of heavy episodic drinking (group 3) relative to low-risk drinking (group 2) and the presence of problem drinking relative to low-risk drinking (group 2). Analyses used STATA 12 (StataCorp, College Station, TX).

## Results

Of the 1200 students approached to take part in the study, 1000 (83%) completed the questionnaires. The mean age was 22.3 (SD=2.36) and the majority of the participants were male (60%). The main characteristics of the study population are presented in Table 1. The prevalence estimates of any alcohol use pattern, low-risk drinking, heavy episodic drinking and problem drinking were 31%, 17.3%, 4.5% and 8.9% respectively.

**Table 1:** simple logistic regression model – factors associated with any alcohol use pattern among university students

Characteristic	Total (N=1000) n (%)	Any alcohol use (N=307) n(%)	No alcohol use (N=693) n(%)	Unadjusted odds ratio 95%CI
**Age category**				
<21yrs	184 (18.40)	42 (13.68)	142 (20.49)	1 1.62(1.11-2.36)^**^
≥21yrs	816 (81.60)	265 (86.32)	551 (79.51)	
**Gender**				
Females	401 (40.10)	101 (32.90)	300 (43.29)	1
Males	599 (59.90)	206 (67.10)	393 (56.71)	1.55(1.17 – 2.06)^**^
**Marital status**				
Married	97 (9.70)	24 (7.82)	73 (10.53)	1
Single	903 (90.30)	283 (92.18)	620 (89.47)	1.40(0.85 – 2.24)
**Year of study**				
1^st^ and 2^nd^ year	465 (46.50)	131 (42.67)	334 (48.20)	1
3^rd^ – 5^th^ year	535 (53.50)	176 (57.33)	359 (51.80)	1.25(0.95 – 1.64)
**Type of scholarship**				
Government	352 (35.20)	112 (36.48)	240 (34.63)	1
Private	648 (64.80)	195 (63.52)	453 (65.37)	0.92(0.69 – 1.22)
**Work status**				
Unemployed	889 (88.90)	270 (87.95)	619 (89.32)	1
Employed	111 (11.10)	37 (12.05)	74 (10.68)	1.14(0.75 – 1.74)
**Physical health status**				
No physical illness	966 (96.60)	296 (96.42)	670 (96.68)	1
Has a physical illness	34 (3.40)	11 (3.58)	23 (3.32)	1.08(0.52 – 2.24)
**Mental health service use**				
No	564 (56.40)	150 (48.86)	414 (59.74)	1
Yes	436 (43.60)	157 (51.14)	279 (40.26)	1.55(1.20 – 2.04)^**^
**Suicidal ideation**				
Absent	786 (78.60)	237 (77.20)	549 (79.22)	1
Present	214 (21.40)	70 (22.80)	144 (20.78)	1.12(0.81 – 1.57)
**Depression symptoms**				
Absent	695 (69.50)	206 (67.10)	489 (70.56)	1
Present	305 (30.50)	101 (32.90)	204 (29.44)	1.20(0.88 – 1.57)
**High levels of academic stress**				
Absent	592 (59.20)	205 (66.78)	387 (55.84)	1
Present	408 (40.80)	102 (33.22)	306 (44.16)	0.63(0.48 – 0.83)^**^
**Recent grade point average**				
≤3.5	403 (40.30)	121 (39.41)	282 (40.69)	1
>3.5	597 (59.70)	186 (60.59)	411 (59.31)	1.05(0.80 – 1.40)

** P-value ≤0.05

Variables associated with any alcohol use pattern in the unadjusted analyses are shown in Table 2. Results from multivariate analysis indicate that gender (OR=1.52, 95%CI (1.14-2.04)), age greater than 25 years (OR=1.45, 95%CI (1.07-1.95)), depression symptoms (OR=1.48, 95%CI (1.08-2.01)), high academic stress levels (OR=0.56, 95%CI (0.41-0.75)) and mental health service use (OR=1.35, 95%CI (1.02-1.79)) were independently associated with any alcohol use pattern. The final model demonstrated satisfactory goodness-of-fit (Hosmer-Lemeshow χ^2^=10.8; P-value=0.21).

**Table 2:** multivariate logistic regression model – factors independently associated with any alcohol use pattern among university students

Variable	Adjusted odds ratio	95%CI	p-value
≥21 years	1.56	1.06 – 2.30	0.023
Male gender	1.50	1.12 – 2.02	0.007
High levels of academic stress	0.55	0.41 – 0.74	0.000
Mental health service use	1.51	1.14 – 1.99	0.003
Depression symptoms	1.48	1.09 – 2.02	0.011

The results of the unadjusted multinomial regression analysis are shown in Table 3. Results from the adjusted multinomial regression (Table 4) indicate that, in comparison to low-risk drinkers those who never used alcohol were significantly more likely to report high academic stress levels, less likely to be more than 25 years old and more likely to report suicidal ideation. In comparison to low-risk drinkers, heavy episodic drinkers were more likely to report high academic stress levels. In comparison to low-risk drinkers, problem drinkers were significantly more likely to be males and to have depression symptoms.

**Table 3:** unadjusted multinomial regression analysis - factors associated with specific alcohol use patterns among university students

Variables	No drinking OR (95% CI)	Heavy episodic drinking OR (95% CI)	Problem drinking OR (95% CI)
≥21 years	0.56(0.34 - 0.92) ‡	0.80(0.31 - 1.98)	0.85(0.41 - 1.78)
Male gender	0.77(0.55 - 1.08)	1.17(0.59 - 2.35)	1.79(1.01 - 3.17) ‡
Most recent GPA >3.5	0.86(0.61 - 1.21)	1.17(0.59 - 2.35)	0.66(0.39 - 1.10)
≥3 years of study	0.75(0.53 - 1.04) †	0.87(0.45- 1.68)	0.85(0.51 - 1.42)
Single marital status	0.52(0.26 - 1.03) †	0.63(0.19 - 2.11)	0.48(0.19 - 1.21)
Sponsor	1.22(0.87 - 1.72)	1.6(0.78 - 3.25)	1.21(0.71 - 2.06)
Has employment	1.03(0.6 - 1.77)	2.15(0.9 - 5.18) †	1.09(0.48 - 2.47)
Has physical Illness	1.95(0.58 - 6.56)	4.05(0.79 - 20.78) †	3.37(0.79 - 14.45) †
Mental health service use	0.71(0.59 - 2.06) ‡	1.01(0.53 -1.95)	1.42(0.85 - 2.38)
Depression symptoms	1.26(0.86 - 1.85)	1.23(0.59 - 2.55)	3.09(1.80 - 5.31) ‡
High levels of academic stress	2.12(1.47 - 3.06) ‡	1.96(1.01 - 3.87) ‡	1.82(1.06 - 3.12) ‡

Table shows comparisons between students with specific alcohol use patterns and the low-risk drinkers; the reference group not included in the table; ‡ P-value ≤0.05; † 0.05 < P-value <0.10

**Table 4:** adjusted multinomial regression analysis - factors associated with specific alcohol use patterns among university students

Variables	No drinking OR (95% CI)	Heavy episodic drinking OR (95% CI)	Problem drinking OR (95% CI)
≥21 years	0.58(0.34 - 0.99) ‡	0.72(0.26 - 2.00)	0.72(0.31 - 1.64)
Male gender	0.84(0.56 - 1.22)	1.29(0.62 - 2.70)	2.25(1.22 - 4.16) ‡
≥3 Years of study	0.83(0.74 - 1.10)	0.86(0.41 - 1.82)	0.92(0.51 -1.66)
Single marital status	0.51(0.25 - 1.02) †	0.74(0.21 - 2.56)	0.55(0.21 - 1.43)
Has employment	0.99(0.57 - 1.75)	1.97(0.79 - 4.90)	0.96(0.40 - 2.26)
Has physical illness	1.41(0.41 - 4.89)	3.20(0.59 - 17.50)	2.23(0.49 - 10.01)
Mental health service use	0.75(0.53 - 1.05) †	1.06(0.54 - 2.07)	1.50(0.87 - 2.51)
Depression symptoms	0.95(0.63 - 1.43)	1.01(0.47 - 2.20)	3.14(1.75 - 5.62) ‡
High levels of academic stress	2.19(1.49 - 3.23) ‡	1.90(0.93 - 3.89) †	1.24(0.69 - 2.22)
Most recent grade point average	0.98(0.68 - 1.41)	1.47(0.71 - 3.02)	0.82(0.48 - 1.42)

Table shows comparisons between students with specific alcohol use patterns and those who are low-risk drinkers (the reference group not included in the table); ‡ P-value ≤ 0.05; † 0.05 < P-value <0.10

## Discussion

Overall, the study found substantial prevalence rates of mal-adaptive alcohol use patterns. Heavy episodic drinking and problem drinking were estimated at 4.5% and 8.9% respectively. These rates are low compared to other studies done; for example, a study done by Sebena *et al*. among 5 European countries in 2012 found problem drinking to range between 11.8% to 22.1% while high frequency drinking at 12.2% to 59.3% [19]. Whereas a study done by Dyrbye *et al*. in 2004 found binge drinking to be at 14.4% [5]. Any alcohol use was more in males than in females and more in individuals aged 21 years and above. Our findings conquer with other studies done among university students which found alcohol use to be correlated with male gender and older age [11,20]. Factors like low response to alcohol effects, greater peer alcohol use effect and socialization into traditional gender roles have been identified as reasons for higher rates of alcohol use among males [21,22].

Interestingly, individuals who used alcohol were significantly less likely to report high levels of academic stress compared to non-drinkers; however, were more likely to have significant depression symptoms and more likely to use mental health services. This could be explained from the tension reduction theory approach, where alcohol a known stress reliever makes alcohol drinkers less likely to report academic stress compared to non-drinkers who may not be having any stress relieving mechanisms. However, the observation that those who reported alcohol use being more likely to use mental health service is a positive sign that availing such support services would easily be utilized.

In relation to academic stress, several studies of stress and substance use among college student populations provide evidence that stress motivates alcohol consumption. Students experiencing higher levels of stress tend to use alcohol and other substances at higher levels and have a higher number of substance-related problems [23,24]. In contrast to these reports, our study results showed that any alcohol use was associated with low stress levels.

Further, in comparison to low-risk drinkers, those students who reported to have never used alcohol had higher levels of academic stress. This finding supports previous studies that have shown moderate consumption of alcohol to have positive influences on physical and mental health, like reduced stress and improving cardiovascular health [25,26]. Consistent with past studies among college students [27,28], our findings confirm the positive association between depressive symptoms and problem drinking among university students.

Problem drinking can be a consequence of depressive symptoms [8], but the relationship works probably in both ways, so that drinking may lead to depressive symptoms [29]. This could probably be explained by the fact that the majority of these problem drinkers reported to have been in a relationship. Thus, social stressors could explain the association between depressive symptoms and problem drinking.

## Conclusion

We confirmed the previously proposed association between depressive symptoms and problem drinking in a culturally different sample of African university students. At the same time, we demonstrated that low-risk drinking may have potential mental health and academic benefits. These findings should be taken into account when developing prevention programs for problem drinking among students. The policy recommendation for addressing problem drinking should include improvement of mental health and development of coping mechanisms. Many students can “feel down” sometimes. For young adults, maladaptive coping mechanisms e.g. drug or alcohol use are common when dealing with social and emotional problems. Such coping strategies are ineffective and provide only immediate relief from stressful situations and may even exacerbate the problems that the person is currently experiencing. Talking openly, having appropriate social support and adequate coping skills can help prevent the transformation of periods of sadness to more severe depression. Prevention programs should directly target specific risk factors (e.g. perceived stress, depressive symptoms) that impact on psychological well-being and focus on implementation programs that teach adaptive coping responses and problem-solving skills so that they can effectively handle problems and stressors that typically characterize university students´ lives.

### What is known about this topic

- University students are at risk of problematic alcohol use;
- Problematic alcohol use affects university students´ academic performance;
- Problematic drinking is associated with mental health problems like depression.

### What this study adds

- High levels of binge drinking were found among the employed/working university students;
- Low-risk drinking was associated with higher academic performance;
- High levels of academic stress were found among non-drinkers.
